# To evaluate the effectiveness of health care ethics consultation based on the goals of health care ethics consultation: a prospective cohort study with randomization

**DOI:** 10.1186/1472-6939-15-1

**Published:** 2014-01-03

**Authors:** Yen-Yuan Chen, Tzong-Shinn Chu, Yu-Hui Kao, Pi-Ru Tsai, Tien-Shang Huang, Wen-Je Ko

**Affiliations:** 1 Department of Social Medicine, National Taiwan University College of Medicine, Taipei, Taiwan; 2 Department of Medical Education, National Taiwan University Hospital, Taipei, Taiwan; 3 Department of Primary Care Medicine, National Taiwan University College of Medicine, Taipei, Taiwan; 4 Department of Sociology, Iowa State University, Ames, IA 50011, USA; 5 Department of Traumatology, National Taiwan University Hospital, Taipei, Taiwan; 6 Department of Internal Medicine, National Taiwan University Hospital, Taipei, Taiwan; 7 Department of Surgery, National Taiwan University Hospital, Taipei, Taiwan

**Keywords:** Health care ethics consultation, Effectiveness, Randomization, Intention-to-treat

## Abstract

**Background:**

The growing prevalence of health care ethics consultation (HCEC) services in the U.S. has been accompanied by an increase in calls for accountability and quality assurance, and for the debates surrounding why and how HCEC is evaluated. The objective of this study was to evaluate the effectiveness of HCEC as indicated by several novel outcome measurements in East Asian medical encounters.

**Methods:**

Patients with medical uncertainty or conflict regarding value-laden issues, and requests made by the attending physicians or nurses for HCEC from December 1, 2009 to April 30, 2012 were randomly assigned to the usual care group (UC group) and the intervention group (HCEC group). The patients in the HCEC group received HCEC conducted by an individual ethics consultant. Data analysis was based on the intention-to-treat principle. Mann–Whitney test and Chi-squared test were used depending on the scale of measurement.

**Results:**

Thirty-three patients (53.23%) were randomly assigned to the HCEC group and 29 patients were randomly assigned to the UC group. Among the 33 patients in the HCEC group, two (6.06%) of them ultimately did not receive a HCEC service. Among the 29 patients in the UC group, four (13.79%) of them received a HCEC service. The survival rate at hospital discharge did not differ between the two groups. Patients in the HCEC group showed significant reductions in the entire ICU stay and entire hospital stay. HCEC significantly facilitated achieving the goal of medical care (*p* < .01). Furthermore, patients in the HCEC group had a shorter ICU stay and shorter hospital stay after the occurrence of medical uncertainty or conflict regarding value-laden issues than those in the UC group.

**Conclusions:**

Our findings demonstrated that HCEC were associated with reduced consumption of medical resources as indicated by shorter entire ICU stay, entire hospital stay, and shorter ICU and hospital stay after the occurrence of the medical uncertainty or conflict regarding value-laden issues. This study also showed that HCEC facilitated achieving a consensus regarding the goal of medical care, which conforms to the goal of HCEC.

## Background

Health care ethics consultation (HCEC) has been applied to clinical practice for several decades. In 1998, the Report of the American Society for Bioethics and Humanities defined HCEC as “a service provided by an individual or a group to help patients, families, surrogates, health care providers, or other involved parties to address uncertainty or conflict regarding value-laden issues that emerge in healthcare” [1].

In 1983, Youngner et al. reported that HCEC was conducted by a hospital ethics committee for approximately 1 case per year, and only 1% of the hospitals with more than 400 beds in the U.S. had a hospital ethics committee [2]. By the end of the 1990s, 93% of hospitals had a hospital ethics committee providing HCEC services. Each hospital ethics committee performed an average of 8.1 formal and 4.3 informal ethics consults per year [3]. Fox et al. recently examined the prevalence of HCEC in the U.S., and reported that, in a random sample of 600 U.S. hospitals, the median number of ethics consults performed per year was three. All the hospitals with more than 400 beds in the U.S. provided HCEC [4]. Johnson et al. also reported that requests for HCEC grew steadily from 2000 to 2008 [5]. According to these studies, the demand for HCEC to resolve ethical conflicts and difficulties in clinical practice has increased tremendously.

The growing prevalence of HCEC services in the USA has been accompanied by an increase in calls for accountability and quality assurance [6-10], and for the debates surrounding why and how HCEC is evaluated [11-14]. Fox et al. enumerated four measurable outcomes for evaluating the effectiveness and quality of HCEC: ethicality; satisfaction; education; and resolution of conflicts [10]. To date, no research study has reported an instrument to measure ethicality. Satisfaction reported by different parties involved in HCEC seems to be the easiest way among the four measurable outcomes, and satisfaction was most frequently used to measure the effectiveness and quality of HCEC [11,12,15,16]. Nevertheless, a potential lack of direct association of satisfaction with the quality of HCEC has been a major concern [17]. For example, it may be that the quality of a CEC is so good that it resolves conflicts and facilitates the decision-making. Patients/surrogates may not be satisfied whatsoever because the decision does not conform to what they desired.

Education is the third measurement used to evaluate the effectiveness and quality of HCEC. Education as a goal of CEC is particularly for health care workers [10]. Studies by La Puma et al. and by Orr et al. reported that the majority of participants in their studies agreed that education helps address ethical problems [15,18]. Although Fox et al. suggested evaluating group level educational outcomes of HCEC is more productive than at an individual case level [10], no instrument has been developed for evaluating the group level educational outcomes. The fourth measurable outcome for HCEC is the resolution of conflicts. Few studies have focused on assessing the resolution of ethical conflicts attributed to HCEC services.

HCEC is still in its infancy in East Asian countries, as well as in Taiwan. In 2008, Fukuyama et al. reported the first small team HCEC services started in October 2006 in Japan, which was also the first formal HCEC published in an academic international journal from East Asia [19]. Until now, there is no formal report published in academic international journals regarding HCEC services in the health care institutions in Taiwan. The objective of this study was to evaluate the outcomes of HCEC. We expected that HCEC positively influence patients’ outcomes as indicated by several dependent variables we newly proposed as compared to prior studies.

## Methods

### Study design

This study was conducted in three surgical intensive care units in National Taiwan University Hospital. Patients with medical uncertainty or conflict regarding value-laden issues, and requests made by the attending physicians or nurses for HCEC from December 1, 2009 to April 30, 2012 were randomly assigned, to the usual care group (UC group) and the intervention group (HCEC group). The patients in the UC group did not receive HCEC, but still received usual care such as family meeting, consultation to social workers and so on, which were considered appropriate by the health care team. The patients in the HCEC group received HCEC conducted by an individual ethics consultant. If a case was assigned to the HCEC group but the attending physician did not want to receive HCEC, the preference of not receiving HCEC was honored. If a case was assigned to the UC group but the attending physician wanted to receive HCEC, the preference of receiving HCEC was honored.

HCEC can be conducted by a hospital ethics committee, a small group of ethics consultants, or an individual ethics consultant [1]. In our study, we conducted HCEC by individual ethics consultants. The qualifications, skills and knowledge of an individual ethics consultant have been proposed by Aulisio et al. [20]. Our individual ethics consultants all have doctoral degrees, received more than a decade of training in clinical medicine, and completed more than 20 hours of clinical ethics educational courses per year. All ethics consultants, while conducting HCEC, were strongly encouraged to follow the suggestions proposed by Aulisio et al. [20]:

1) Gather relevant data.

2) Clarify relevant concepts.

3) Clarify related normative issues.

4) Help to identify a range of morally acceptable options within the context.

5) Facilitate consensus among involved parties.

### Data collection

Data was concurrently and retrospectively collected from the review of medical records and HCEC records. We collected independent variables such as patient demographics and severity of illness as indicated by the total of Elixhauser comorbidity measures. Although the total of Elixhauser comorbidity measures is not an acute severity score, it is the only severity of illness which can be collected in all the three intensive care units. Three groups of outcome measurements were also collected: first, patient status at hospital discharge; second, consumption of medical resources as indicated by length of ICU stay, length of hospital stay, post-conflict length of ICU stay, and post-conflict length of hospital stay; and third, whether a consensus regarding patient care was achieved, as an indicator of whether the goal of HCEC was achieved. A consensus regarding the goal of medical care was achieved in the HCEC group if any of the morally acceptable options suggested by the individual ethics consultant was followed, and in the UC group if patients/family members and health care team members agreed on any options for the goal of medical care. Health care team members (i.e., the nurse in charge of the patient, head nurse, or the primary care resident) were contacted about whether there was a consensus on the goal of medical care after the onset of medical uncertainty or conflict regarding value-laden issues in the UC group, or after the HCEC was done in the HCEC group.

### Intention-to-treat principle

Data analysis was based on the intention-to-treat principle, implying that the comparison of variables is based on the initial treatment assignment and not on the treatment eventually received. For example, if a patient with medical uncertainty or conflict regarding value-laden issues was randomly assigned to the HCEC group, but ultimately did not receive HCEC service, the patient was still retained in the HCEC group when the data were analyzed.

### Statistical analysis

Our analysis examined univariate characteristics (central tendency, dispersion, and distribution) and bivariate relationships (correlations). These exploratory techniques were based on proportions (categorical variables) and medians (variables measured on an interval scale). The appropriate non-parametric tests, such as Mann–Whitney test and the Chi-squared test, were used depending on the scale of measurement. All statistical analyses were executed using STATA/MP 11.0 for Windows PC. This study was approved by the Research Ethics Committee in National Taiwan University Hospital.

## Results

During data collection period, a total of 62 patients with the medical uncertainty or conflict regarding value-laden issues and requests for HCEC service were collected. The number of issues for each request ranged from one to three, with a median of 2 ± 0.63 (median ± standard deviation) issues. The three leading issues for requesting HCEC service by the physicians were disagreement between health care team and family member (n = 14), cardiopulmonary resuscitation/do-not-resuscitate (n = 9), and withholding/withdrawing life-supporting treatment (n = 8). The three leading issues for requesting HCEC service by the nurses were disagreement between health care team and family member (n = 23), withholding/withdrawing life-supporting treatment (n = 15), and disagreement between health care team members (n = 13) (Table 1).

**Table 1:** Issues which health care workers sought assistance with during data collection period

**Issues**	**Request for HCEC made by**		**Total**
	**Physicians**	**Nurses**	
Disagreement between health care team and family members	14	23	37 (59.68%)
Withholding/Withdrawing life-supporting treatment	8	15	23 (37.10%)
Cardiopulmonary resuscitation/Do-not-resuscitate	9	12	21 (33.87%)
Unclear goal of medical care	7	11	18 (29.03%)
Disagreement between health care team members	3	13	16 (25.81%)
Individual autonomy/Family autonomy	0	2	2 (3.23%)
Treatment refusal	2	0	2 (3.23%)
Legal issues	1	1	2 (3.23%)
Complementary and alternative medicine	0	1	1 (1.61%)
Hospice/Palliative Care	0	1	1 (1.61%)
Negligence	1	0	1 (1.61%)
Euthanasia	1	0	1 (1.61%)
Surrogacy	0	1	1 (1.61%)

*Abbreviation List*: *HCEC*, health care ethics consultation.

Among the 37 requests for consultation due to the issue of disagreement between health care team and family member, 14 (37.84%) were made by the patient’s attending physician. By comparison, only three (18.75%) of the 16 requests for consultation due to the issue of disagreement between health care team members were made by the patient’s primary care nurse or head nurse (Table 1).

**Figure 1:**
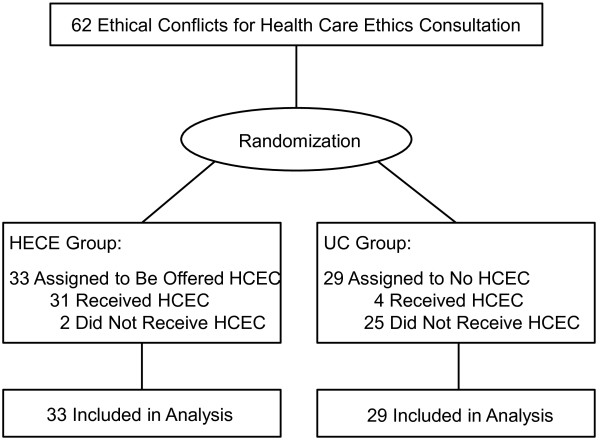
Patient flow.

Thirty-three patients (53.23%) were randomly assigned to the HCEC group and 29 patients were randomly assigned to the UC group. Among the 33 patients in the HCEC group, two (6.06%) of them ultimately did not receive a HCEC service. Among the 29 patients in the UC group, four (13.79%) of them received a HCEC service (Figure 1). Among the total of 62 requests for HCEC service, 25 (40.32%) were made by the attending physician and 37 by the primary care nurse or the head nurse.

In the HCEC and UC groups, 26 (78.79%) and 21 (72.41%) patients died at hospital discharge, respectively (*p* = .56). A total of 28 patients (84.85%) assigned to the HCEC group reached a consensus regarding the goal of medical care after completing the HCEC service, and seven patients (24.14%) in the UC group reached a consensus (*p* < .01). Even considering the crossover and comparing the patients who actually received HCEC with those who actually did not receive HCEC, the 35 patients who actually received HCEC service were more likely to reach a consensus regarding the goal of medical care than the 27 patients who did not (*p* < .01) (Table 2).

**Table 2:** Comparison of characteristics between patients assigned to HCEC group and patients assigned to UC group

	**HCEC Group N = 33**	**UC Group N = 29**	***p *** **value**
***Patient Characteristics***			
**Age on admission, year**	51 ± 20.82	46 ± 23.29	0.53
**Gender**			0.66
Female	12 (36.36%)	9 (31.03%)	
Male	21 (63.64%)	20 (68.97%)	
**Marital status**			
Married	18 (54.55%)	13 (44.83%)	0.45
Unmarried	15 (45.45%)	16 (55.17%)	
**Educational level**			0.52
University of Higher	14 (42.42%)	10 (34.48%)	
Middle school or Lower	19 (57.58%)	19 (65.52%)	
**Religion**			0.81
Buddhism/Daoism	17 (51.52%)	15 (51.72%)	
Christian/Catholics	2 (6.06%)	3 (10.34%)	
Others	14 (42.42%)	11 (37.93%)	
**Elixhauser comorbidity measures**	2 ± 1.80	2 ± 1.54	0.47
**HCEC requested**			0.72
Nurse	19 (57.58%)	18 (62.07%)	
Attending Physician	14 (42.42%)	11 (37.93%)	
***Outcome Data***			
**Discharge status**			0.56
Dead	26 (78.79%)	21 (72.41%)	
Survived	7 (21.21%)	8 (27.59%)	
**Total ICU stay, day**	17 ± 17.26	30 ± 37.50	0.05
**Total hospital stay, day**	25 ± 35.80	70 ± 42.05	< 0.01
**Post-conflict ICU stay, day**^***a***^	6 ± 13.87	20 ± 23.86	< 0.01
**Post-conflict hospital stay, day**^***b***^	7 ± 18.52	21 ± 25.02	< 0.01
**The average of ethical issues**	2 ± 0.63	2 ± 0.58	0.42
**Consensus reached**			< 0.01
No	5 (15.15%)	22 (75.86%)	
Yes	28 (84.85%)	7 (24.14%)	

*Abbreviation list*: *HCEC*, health care ethics consultation; *UC*, usual care; *ICU*, intensive care unit.

Continuous variables of the two groups were compared using Mann-Whitney test, and the test results were shown as median ± standard deviation. Categorical variables of the two groups were compared using the Chi-squared test, and the test results were shown as number (percentage).

^*a*^ “Post-conflict ICU stay, day” means the length of ICU stay by days after the medical uncertainty or conflict regarding value-laden issues happened.

^*b*^ “Post-conflict hospital stay, day” means the length of hospital stay by days after the medical uncertainty or conflict regarding value-laden issues happened.

Patients in the HCEC group showed significant reductions in the entire ICU stay and entire hospital stay. In addition, patients in the HCEC group had a shorter ICU stay and shorter hospital stay after the occurrence of medical uncertainty or conflict regarding value-laden issues than those in the UC group (Table 2).

## Discussion

### Main findings

Examining the effectiveness of HCEC demonstrated that HCEC was associated with achieving a consensus regarding the goal of medical care, shorter length of entire ICU and hospital stay, and shorter length of ICU and hospital stay after patients encountering medical uncertainty or conflict regarding value-laden issues. In addition, patients in the HCEC group did not have a higher mortality rate than those in UC group at hospital discharge.

### Strengths and limitations

This is the first study to evaluate the effectiveness of HCEC conducted in East Asian medical encounters, where the core value of medical decision-making may be distinct from North America/Europe [21]. Our individual ethics consultants were encouraged to follow the way of conducting HCEC suggested by the scholars from North America [20]. In addition, we proposed several novel outcome measurements to evaluate the effectiveness of HCEC, such as whether a consensus regarding the goal of medical care was achieved, the length of ICU stay and hospital stay after the occurrence of ethical conflicts. Whether a consensus was achieved is a better outcome measurement than most of the outcome measurements reported in the literature because it conforms to the goals of HCEC [1]. Lastly, our study used randomization and intention-to-treat principle to evaluate the effectiveness of HCEC [11,12]. Although there are some concerns about incomplete double blindness [14], randomization and the principle of intention-to-treat are still considered the most rigorous study design to evaluate the effectiveness of HCEC.

Our study has limitations. First, this is a single center study. The generalizability of the study results may be limited. Second, the differences observed in the outcome measurements could be overestimated because the involved parties in this study were not blinded regarding the HCEC service. If health care team members in the HCEC group reflected greater enthusiasm than those in the UC group because they knew that a HCEC service is being conducted for their patient, the differences observed in the outcome measurements might be partly associated with the greater enthusiasm. Third, although we evaluated the effectiveness of HCEC using one of the goals of HCEC as the outcome measurement, whether the other goals of HCEC were achieved was not examined. Fourth, some of the readers for this paper might be concerned that this is a self-reporting success because only the health care team members were inquired regarding “whether a consensus was reach”. However, until now, the health care team members still do not know that we evaluate the effectiveness of HCEC using “whether a consensus was reached” as an outcome measure.

### Ethical issues for requesting health care ethics consultation

Several studies have reported the ethical issues which the patient or health care team members encountered for requesting HCEC. La Puma et al. reported that 49% of the cases requested HCEC for assistance with withdrawing or withholding life-supporting treatments, 37% for resuscitation issues, and 31% for legal issues [16]. Another study conducted by La Puma et al. showed that 74% of the cases requested HCEC for the decisions to forgo life-supporting treatments, 46% for resolving disagreement, and 30% for assessing patient competence for decision-making [15]. A recent study conducted by Johnson et al. reported that the requesters sought assistance with end-of life issues in 47% of the cases, in 41% of cases for shared decision-making, and in 14% of cases for professionalism [5].

Most of the HCEC requesters sought assistance with more than one issue. In addition, the majority of requests made for HCEC surrounded end-of-life issues, e.g. withdrawing or withholding life-supporting treatments, cardiopulmonary resuscitation/do-not-resuscitate, and disagreement. These studies reflected the educational needs for health care workers in resolving medical uncertainty or conflict regarding value-laden issues. They also demonstrated the issues that an ethics consultant should be familiar with, and capable of resolving.

### Empowerment for requesting health care ethics consultation

According to Johnson et al., most of the requests for HCEC were placed by house officers (63%), nurses (12%), and attending physicians (11%) [5]. Our study showed that 37 (59.68%) of the 62 requests for HCEC were made by the primary care nurses or head nurses, and the remaining were made by the attending physicians. Both studies revealed that non-MDs participate in requesting HCEC service, particularly in our study, in which a higher percentage of HCEC requests were made by non-MDs than in the Johnson study. This may be associated with the fact that the nurses in the three ICU setting were encouraged to assist patients by requesting HCEC services.

We also identified that the physicians were more likely to request HCEC when encountering the issue of disagreement between health care team and family members than the issue of disagreement between health care team members. For the 37 disagreements between health care team and family members, 14 (37.84%) of them were requested by the attending physicians. For the 16 disagreements between health care team members, only three (18.75%) were requested by the attending physicians. These findings may imply that nurses are more likely than attending physicians to identify disagreements between health care team members as a problem, and that the attending physician may not see the disagreement as a problem or not be aware of the disagreement.

### Appropriateness of outcome measurements

Numerous empirical studies have been initiated because of concerns related to accountability and quality assurance in HCEC. Many of these studies reported findings on physician satisfaction [11,12,15,16], and physician’s perception of clarifying ethical issues, educating the health care team, making clinical decisions with confidence, and in patient management [18]. However, the satisfaction or perception of the parties involved may be influenced by factors not associated with the quality of the HCEC conducted [17,22]. For example, a physician may be satisfied with the HCEC service provided because his/her suggestion is adopted by the ethics consultant and not because of the quality of the HCEC service.

Schneiderman et al. reported that, for those who did not survive to hospital discharge, HCEC was significantly associated with lower cost, shorter ICU stays, shorter hospital stays, and less use of life-supporting treatments. The studies also showed that HCEC was beneficial to patients who did not survive to hospital discharge [11,12,23].

The appropriateness of randomized controlled trials in evaluating HCEC has been a concern because these trials are not double-blinded [14]. Moreover, researchers have argued that monetary saving should not be included as an outcome measurement to evaluate the effectiveness of HCEC because lowering costs is not one of the goals of HCEC [13]. Rigorous scientific research results supporting the effectiveness and quality of HCEC appear to lag far behind the rapid growth of HCEC and concerns about its accountability and quality assurance.

The effectiveness of HCEC is evaluated based on measurable outcomes that are consistent with the intended goals of HCEC [17]. If the intended goals of HCEC are followed, the quality of HCEC is satisfactory. Therefore, to determine the outcome measurements required to evaluate the effectiveness and quality of HCEC, we can refer to the established goals of HCEC [9].

The Society for Health and Human Values-Society for Bioethics Consultation Task Force on Standards for Bioethics Consultation organized a consensus panel with professionals and experts [1]. The goals of HCEC were proposed by the task force. As pointed out by the task force, we noted that whether a consensus regarding the goal of medical care was achieved is a critical outcome measurement for evaluating the effectiveness of HCEC. Therefore, whether a consensus regarding the goal of medical care was achieved was examined and compared between the HCEC group and UC group in this study. We identified that HCEC services facilitated achieving a consensus regarding the goal of medical care effectively, thus conforming to the goals of HCEC proposed by the American Societies for Bioethics and Humanities.

The results of our study agree with those of several previous studies showing, for example, that HCEC is associated with short lengths of entire ICU stay and entire hospital stay [11,12]. However, the entire ICU stay and hospital stay in our study were considerably longer than those reported by Schneiderman et al. Therefore, we examined the outcomes of HCEC by using the length of ICU stay and hospital stay after the occurrence of medical uncertainty or conflict regarding value-laden issues, which more directly measured the influence of HCEC than the length of entire ICU stay and entire hospital stay.

### Cultural differences in conducting health care ethics consultation

Our ethics consultants were encouraged to conduct HCEC following the ethics facilitation approach as proposed by Aulisio et al. Part of the rationale to support this approach to conducting HCEC in the U.S., according to Aulisio et al. [20], are that the U.S. is a pluralistic society, and the main societal value is individual autonomy. To honor each moral stakeholder from different racial/ethnic backgrounds, and also to uphold the societal value of respecting individual autonomy, the voice of each moral stakeholder surrounding the ethical conflict should be heard, and his/her preferences should be respected. Therefore, ethics facilitation approach for conducting HCEC is highly suggested in the U.S.

However, the ethics facilitation approach to conduct HCEC in the medical encounters in Taiwan might be of concern because individual autonomy may not be the main societal value. For several thousand years, Confucian philosophy has deeply influenced societal values, and ethical considerations in East Asian countries such as Taiwan [24]. One phenomenon rooted in Confucian philosophy highlighting the difference between East Asian countries and North America/Europe is the locus of authority in decision-making: North America/Europe demands and promotes the value of individual autonomy; East Asian countries typically honor and uphold the value of family autonomy [25]. Although the ongoing westernization of East Asian biomedical ethics in Taiwan is convincing, family autonomy seems to remain as the main societal value [21]. As such, the appropriateness of applying the ethics facilitation approach to conducting HCEC in Taiwan’s medical encounters should be further deliberated.

Before this study was conducted, HCEC services were not formally announced to National Taiwan University Hospital. There were only few formal and informal HCEC services conducted by two individual ethics consultants who had several years of clinical ethics training as well as medical training. Currently, given that HCEC services have been formally announced to National Taiwan University Hospital and the institutional supports in place for HCEC services, a group of individual ethics consultants (composed of physicians, nurses and social workers) are conducting daily HCEC services, and, as a result, healthcare professionals’ requests for HCEC are dramatically increasing.

## Conclusions

We conducted this study to examine the effectiveness and quality of HCEC in East Asian medical encounters by following the suggestions of conducting HCEC proposed by the scholars from the U.S. Our findings demonstrated that HCEC reduced the consumption of medical resources as indicated by shorter entire ICU stay, entire hospital stay, and shorter ICU and hospital stay after the occurrence of the medical uncertainty or conflict regarding value-laden issues. This study also showed that HCEC facilitated achieving a consensus regarding the goal of medical care, which conforms to the goal of HCEC. Future studies should focus on qualitative approaches to examine HCEC, the appropriateness of ethics facilitation approach in East Asian medical encounters where family autonomy/family-determination is usually highlighted.

## Competing interests

The authors declare that they have no competing interests.

## Authors’ contributions

YC carried out the literature review, study design, statistical analysis and drafted the manuscript. TC carried out the statistical analysis, edited the draft and tables. YK helped with the literature review and statistical analysis. PT helped with study design and data collection. TH helped with drafting the manuscript and coordination. WK participated in the study design. All authors read and approved the final manuscript.

## Pre-publication history

The pre-publication history for this paper can be accessed here:

http://www.biomedcentral.com/1472-6939/15/1/prepub
